# To Wrap or Not? Utility of Anti-reflux Procedure in Infants Needing Gastrostomy Tubes

**DOI:** 10.3389/fped.2022.855156

**Published:** 2022-03-07

**Authors:** Faraz A. Khan, Kelsey Nestor, Asra Hashmi, Saleem Islam

**Affiliations:** ^1^Division of Pediatric Surgery, Department of Surgery, Loma Linda University, Loma Linda, CA, United States; ^2^Division of Pediatric Surgery, Department of Surgery, University of Florida College of Medicine, Gainesville, FL, United States; ^3^Department of Plastic Surgery, Loma Linda University, Loma Linda, CA, United States

**Keywords:** gastrostomy tube, fundoplication, anti-reflux procedure, gastroesophageal reflux, nissen fundoplication

## Abstract

**Purpose:**

Gastrostomy tube (GT) insertion is commonly performed in children with failure to thrive. Pediatric patients' frequently have gastroesophageal reflux (GER) and discerning pathological GER can be challenging. Moreover, there is some evidence that GT insertion may lead to worsening GER and to avoid a subsequent anti-reflux procedure (ARP), though controversial some surgeons advocate considering an ARP concomitantly. The purpose of this report is to assess outcomes in infants who underwent a GT vs. GT with ARP.

**Methods:**

Retrospective review of all infants who had a GT placed at a single institution from 2009–2014. The patients were then divided into two cohorts based on the index operation i.e., GT vs GT with ARP and outcomes compared.

**Results:**

226 operations (104 GT, 122 GT with ARP) were performed. The cohorts were similar in gender, gestational age, race, weight, median age, LOS, and proportion of neurologically impaired patients. Preoperative GER was significantly higher in the GT with ARP cohort (91 vs. 18%). No difference in the rate of immediate complications was noted between the two groups. Postoperative increase in anti-reflux medications was significantly higher in the GT cohort (*p* = 0.01). Post-op GER needing a secondary procedure (ARP or GJ tube) was noted in 21/104 (20%) patients. Those needing an additional procedure vs. those with GT alone were similar in the proportion of patients with pre-op GER, neurologic impairment, type of feeds, and age.

**Conclusion:**

Identifying patients who would benefit from a concomitant ARP remains challenging. A fifth of GT patients needed a subsequent procedure despite most high-risk patients having already undergone an ARP. Since the overall rate of complications remained similar, initial GT approach can be considered reasonable.

## Introduction

A large number of infants aged less than 5 months of age have some degree of gastroesophageal reflux (GER), however this progressively decreases over time to less than 5% of the population by a year of age (1). GER is defined as the passage of gastric contents into the esophagus with or without regurgitation and/or vomiting. It is considered pathologic and referred to as gastroesophageal reflux disease (GERD) when the reflux leads to troublesome symptoms and/or complications, such as esophagitis or stricture (2). Reported symptoms of infant GERD vary widely and may include excessive crying, back arching, regurgitation and irritability. Since these symptoms vary widely by age and are non-specific establishing temporal relationship of these symptoms to reflux events therefore becomes very difficult. This is particularly applicable to non-verbal infants in whom many of these symptoms occur with or without GERD and defining what is considered troublesome becomes challenging which in turn makes establishing a diagnosis of GERD difficult. Physiologic regurgitation and episodic vomiting are frequent in infants, however presence of red flag signs and symptoms and onset of GERD symptoms after the age of 6 months or persistence of symptoms beyond 12 months raises the possibility of alternative diagnoses to infant GER (2). The diagnosis of GERD however remains a primarily a clinical one which can be strengthened by additional diagnostic investigations aimed mainly to quantify and qualify the nature of GER. While there is a myriad of diagnostic tests such as contrast imaging, biomarkers, esophageal manometry, pH probe studies, esophagogastroduodenoscopy with/without biopsy, scintigraphy etc. used in clinical practice there is no clear established gold standard and in fact several lack sufficient evidence to support use in the evaluation and management of pediatric patients with GERD (2).

Gastrostomy tubes (GT) are frequently used in infants and children with failure to thrive, neurologic issues, developmental delay, and other conditions in whom feeding access is required (3). There is considerable debate whether insertion of a GT increases GER or may precipitate worsening of symptoms related to GER (4). Some previous investigators have reported GER in post GT patients at a rate of 13–28% (5). When post GT insertion pH is measured various studies have reported no significant changes in esophageal pH, (6) while others conclude that some patients post GT insertion had an increase or decrease in esophageal pH. However, a majority of those without pre-existing pathological GERD did not require any further surgical interventions to manage their symptoms (4, 7). There are a number of theories postulated to explain the worsening of the GER post GT insertion. These include an increase in trans diaphragmatic pressure during coughing, type and location of the GT and mechanism of feeding (bolus vs. continuous) (8, 9). This post-operative reflux can be potentially dangerous in patients with impaired swallowing mechanism or inability to protect the airway as it could lead to complications such as aspiration pneumonia (10, 11). The need for a subsequent anti-reflux procedure after GT insertion occurs in 7–16% of patients which while a considerable minority remains a consideration at the index operation, particularly in neurologically impaired children where this rate may be as high as 20% (5, 8). Not only do the tests used to assess pre-operative GER have considerable shortcomings, there is variation in what pre-operative work is done among institutions and pediatric surgeons. One survey of 121 pediatric surgeons reported that 80% required a UGI before anti-reflux procedure and 13% required a pH probe. While surgeons rated their opinion as the most important in deciding to offer a concomitant anti-reflux procedure, both parental and referring physician opinion was highly influential in this decision irrespective of the UGI and pH probe data (12). Concomitantly, over the past decade, there has been a substantial reduction in the number of ARP's in the US (13). The decision to perform a concomitant ARP in patients in need of long-term enteral access remains extremely subjective given the lack of uniform application of preoperative work up to quantify GER and as evidenced by the tremendous variation in the rate of decline in anti-reflux procedures performed in the free-standing children's hospitals in the US (14).

Children less than 1 year old due to the relatively high overall prevalence of GER are a challenging subset of patients, as the ability to reliably distinguish physiological GER from GERD is limited. The purpose of this study was to compare demographics and outcomes of infants initially evaluated for long term enteral feeding access who had GT placement alone vs. those who had GT with a concomitant anti-reflux procedure and identify the rate and characteristics of patients needing a subsequent anti-reflux procedure after GT placement.

## Methods

After obtaining IRB approval, a retrospective review was performed for all patients less than 12 months of age who underwent a gastrostomy tube placement at a tertiary care children's hospital between 2009 and 2014.

### Patient Selection

The inclusion criteria included patients who had the tube placed by any method (open, endoscopic, radiologic, or laparoscopic) and those who had concomitant anti-reflux or other procedures during the same anesthetic. Patients who had the tube placed at an outside facility or were older than 12 months of age at the time of the initial procedure were excluded.

### Definitions and Data

Data collected included demographics, associated anomalies, and prior operations. Specifics included the indications for the procedure, the presence of pre-operative GER, medication use, and other comorbid conditions at the time of the operation. Neurologic impairment was a key comorbid condition included, and was defined as having cerebral palsy due to any cause, hypotonia, or structural brain anomalies with or without hydrocephalus. Similar to what can be surmised by the pediatric surgeon survey data no specific objective investigations to quantify degree of GER at the time of initial evaluation were uniformly performed. Clinical criteria used to decide approach for the index operation namely GT without ARP included ability to tolerate nasogastric tube feeds in the form of a bolus (administered over an hour or less) without increase in spit ups or clinical stigmata of reflux (back arching) and maintenance of growth over a 2-week period. Operative data included technique used (open, laparoscopic, or PEG), concurrent procedures, hospital LOS, and use of anti-reflux medications. Outcome data included post-operative anti-reflux medication use, immediate and long-term mechanical and feeding complications. Specific attention was placed on subsequent need for anti-reflux procedures (fundoplication or conversion to gastrojejunostomy tube).

### Operative Technique

Laparoscopic gastrotomy tubes were performed typically using the Georgeson technique. Open gastrostomy tubes were performed using Stamm technique. Fundoplication procedures were performed by three surgeons and a loose wrap was created without the use of a sizing bougie. The posterior dissection was kept at a minimum unless needed to ensure adequate intra-abdominal length of the esophagus.

### Statistical Analysis

Data analysis was performed using Minitab 17 Statistical Software (2010); State College, PA: Minitab, Inc. (www.minitab.com). Continuous variables were analyzed using student's *t*-test, or the Mann Whitney *U* test for non-parametric data. Categorical variables were analyzed using proportional statistics (Fischer's Exact Test). Significance was inferred at *p* < 0.05.

## Results

A total of 226 patients less than 12 months of age underwent GT with or without a concomitant anti-reflux procedure. Overall, the median age was 3 months (range 0.03–12 months), median gestational age at birth was 37 weeks (range 22–41 weeks), and the median weight at surgery was 4.5 kg (range 1.6–19.4 kg). The male to female ratio was 1:2, and the most common diagnosis was failure to thrive which led to the GT consultation. Majority (69%) of patients underwent laparoscopic procedures, with 23 performed open and 8% percutaneously with endoscopic guidance. The open procedures were performed in patients who had previous abdominal operations such as repair of congenital diaphragmatic hernia with significant adhesions precluding a laparoscopic approach.

On the basis of the index operation, the cohort was then divided into those with a GT only (*n* = 104), and GT with an anti-reflux procedure (ARP) (*n* = 122). Demographics and preoperative characteristics of the two groups are compared in Table 1. There was a significantly higher rate of clinical pre-operative GER recorded in patients in the ARP group (91 ARP vs. 18% GT, *p* < 0.0001). There were no differences in the age (2.75 months GT vs. 2.15 months ARP, *p* = 0.93) or weight (3.9 kg GT vs. 4.25 kg ARP, *p* = 0.54) of the patients at the time of the index operation. The groups were also similar in the proportion of patients with failure to thrive and presence of neurologic impairment (Table 1).

**Table 1:** Comparison of patients with initial GT alone vs. GT with ARP.

	**Gastrostomy tube** **(*n* = 104)**	**Fundoplication + Gastrostomy tube (*n* = 122)**	***p*-value**
Age at index operation (months)	2.75 (0.03–12)	2.15 (0.33–12)	0.93
Male (*n*)	38 (36.5%)	37 (30%)	0.39
Female (*n*)	66 (63.5%)	85 (69.7%)	0.39
Gestational Age (weeks)	36 (22–41)	37 (23–41)	0.43
Weight (kg)	3.9 (1.6-13)	4.25 (2.3–12)	0.54
Failure to Thrive (*n*)	86 (82.7%)	110 (90.1%)	0.11
Pre-operative GER (*n*)	18 (17.3%)	91 (74.6%)	0.0001
Neurological Impairment (*n*)	28 (26.9%)	36 (29.5%)	0.76
Congenital Heart Disease (*n*)	17 (16.3%)	16 (13.1%)	0.57
Increase in Anti-reflux medications (n)	70 (67.3%)	28 (23%)	0.01
Subsequent anti-reflux procedure (*n*)	21 (20%)	n/a	n/a
Complications (*n*)	32 (31%)	25 (20.5%)	0.08

Post-operatively there was an increase in the need for anti-reflux medications (including PPIs, etc.) among patients in both groups, however, the GT alone group had a significantly higher amount of anti-reflux medications post-surgery (67.3 GT vs. 23% ARP, *p* = 0.01) (Table 1). Both groups were similar in complication rates including mechanical (tube leakage, tube migration, etc.), granulation tissue complications, and feeding complications (feeding intolerance, etc.). 21 (20%) patients who underwent GT alone needed a secondary procedure such as conversion to gastrojejunostomy tube or a Nissen fundoplication due to clinically significant reflux at a median of 2 months (range 1–53) after the index operation (Table 1). We studied these patients as a separate cohort (Table 2). This cohort of 21 patients needing a subsequent procedure showed no difference in pre-operative GER compared to the other patients in the index GT cohort. Additionally, both groups of patients were similar in the proportion of patients with neurologic impairment, pre-operative comorbidities, and need for anti-reflux medications pre-operatively. There were three patients with Trisomy 21 in the group needing a subsequent procedure compared to none in the GT alone group, however this did not achieve statistical significance.

**Table 2:** Comparison of GT alone vs. subsequent anti-reflux procedure.

	**Gastrostomy tube alone (*n* = 83)**	**Subsequent anti-reflux procedure** **(*n* = 21)**	***p*-value**
Age at index operation (months)	2.4 (0–12)	3 (0.5–10)	0.41
Weight at index operation (kg)	3.9 (1.6–13)	3.47 (2.1–12)	0.85
Pre-Op GER (*n*)	16 (19.2%)	5 (21.7%)	0.76
Pre-Op GI Meds (*n*)	39 (47%)	12 (57%)	0.47
Neurologic impairment (*n*)	25 (30%)	3 (14.2%)	0.17

## Discussion

Analysis of these data suggests that accurate pre-operative prediction of which patients will need a concomitant anti-reflux procedure at the time of GT insertion remains challenging. These data review the typical day to day clinical decision making when presented with a patient in need of long-term enteral feeding access. As previously discussed a number of investigations are available to qualify GER they are not universally applied to the clinical decision-making pathways and distinction between GER and GERD remains clinical and somewhat arbitrary. A fifth of patients in this cohort needed a secondary anti-reflux procedure (fundoplication or GJ tube insertion) following GT alone, which is significant since most patients presumed to be at high risk of GER at pre-operative evaluation were selected out into the ARP with GT approach at the index operation. Neurologic impairment is frequently reported in the literature to be a potential risk factor for the development of significant GER needing an anti-reflux procedure, however neurological impairment was not found to be independently associated with an increased risk of needing a secondary anti-reflux procedure in the GT alone cohort. Complication rates in both groups however were similar, therefore either approach can be potentially considered when evaluating patients for long term enteral feeding access. Of note none of the patients needing a subsequent fundoplication procedure required a revision of the GT site nor was there any additional difficulty such as higher rate of conversion to an open procedure. It is the authors inference that since overall outcomes are fairly similar regardless of approach at the index operation which when coupled with no significant technical challenges encountered if a subsequent anti-reflux procedure is performed, little is lost with an initial GT strategy alone. Avoiding a subsequent procedure and exposure to another anesthetic would be ideal, however, since identification of the subset of patients most likely to benefit from ARP at the index operation remains elusive GT alone may be a reasonable consideration in the majority of patients. However, there remains a need for identification of more reliable methods to accurately predict failure of a GT alone strategy and need for a subsequent ARP procedure.

Insertion of GT directly into the stomach through the abdominal wall, has been used since the late 1800's to bypass gastrointestinal dysfunction. In children with neurological impairment GT has been used to bypass oral motor dysfunction when conventional treatment (i.e., positioning, therapeutic techniques to facilitate lip closure and swallowing, thickened food and liquid, and extended feeding time) have failed to resolve oro-motor dysfunction and dyscoordination leading to failure to thrive (15, 16). Additionally, GER in children with neurological impairment is frequently seen and the incidence has been reported to range from 14–5% (17, 18). The increased GER seen in children with neurological impairment is multifactorial and is theorized to occur due to impaired motility of the esophagus and the lower esophageal sphincter leading to involuntary retrograde displacement of gastric contents (19, 20). Children with neurological impairment additionally have pathological reasons for increased intra-abdominal pressure such as spasticity of the abdominal musculature, constipation etc. which when combined with considerable amount of time these children may spend in the supine position due to their disability likely contributes to the GER seen (21). Children with neurological impairment with pre-existing GER typically have some worsening of the GER following insertion of GT (22). Since neurologically impaired children with GER are typically challenging to manage medically, some physicians proposed routine use concomitant of anti-reflux procedures with GT (23, 24).

As previously discussed GER can be normally present in infants and discerning physiologic GER from pathological GER can be somewhat challenging. In most children regurgitation or physiologic GER follows a benign course and usually resolves by 12–18 months of age. However, 5–8% of infants may have troublesome pathologic GER which may manifest with complications ranging from mucosal damage to extra-esophageal manifestations such as sleep disturbances, weight loss, chronic respiratory symptoms etc (25). Moreover, there is no gold standard technique available for investigating GER, multiple options are available and used with wide variability between surgeons and institutions (12). Each option to assess severity of GER has its own associated short comings. 24-h pH monitoring is frequently used to quantify the amount of acid exposure to the esophagus, this however does not consistently correlate with severity of symptoms or complication associated with GER (25). Another modality used is multiple intraluminal impedance (MII) which helps measure gastrointestinal motility and is effective at evaluating the temporal relationship between both acid and non-acid reflux and clinical symptoms (26). The combination of pH-metry with MII increases the diagnostic yield of identifying pathological GER and has been shown to correlate with the presence of endoscopically conformed esophagitis (27). However, the application of this technique has not been fully validated in children and at present North American Society for Pediatric Gastroenterology, Hepatology, and Nutrition (NASPGHAN) and the European Society for Pediatric Gastroenterology, Hepatology, and Nutrition (ESPGHAN) guidelines do not support use of MII-pH as a single evaluation modality citing insufficient available evidence (2). Endoscopy with or without biopsy can be helpful in evaluating complications such as Barrett's esophagus, however absence of endoscopically visible or histological changes do not rule out clinically significant pathological GER (28). Survey data suggest that when surgeons evaluate patients for GT insertion, the decision of performing a concomitant ARP is based on the general gestalt of the patient and is not necessarily based on specific test data (12). This approach is likely driven by the poor performance of any tests in correctly identifying patients that would benefit from an anti-reflux procedure.

Anti-reflux procedures include a variety of fundoplication procedures such Nissen, Thal, Toupet, Belsey etc. and insertion of gastrojejunostomy tubes. All fundoplication procedures aim to increase the barrier to acid reflux by a number of mechanisms including increasing the pressure of the lower esophageal sphincter, tightening the crural opening, lengthening the intra-abdominal portion of the esophagus and correcting a hiatal hernia if present (29). Anti-reflux procedures however are associated with a considerable recurrence rate of GER leading to an up to 40% rate of surgical failure (30). Failure of fundoplication and recurrent GER is more commonly seen in children with neurological impairment. Up to 30% patients with neurological impairment experience recurrent GER following an anti-reflux procedure this is in addition to a 59% rate of complications and 1–3% mortality. Complications related to the surgery include but are not limited to gas bloat syndrome, gastric hypersensitivity, dumping syndrome, retching, and dysphagia (31). While data did not show a statistically significant increase in the rate of complications in the ARP group, potential of these complications and the fact that subsequent procedures are not particularly technically challenging supports potentially using GT alone as a reasonable first intervention provided none of the complication of persistent GER haven't already developed. Not surprisingly there has been an almost 3-fold decrease in the number of pediatric fundoplication procedures for GER in the last decade (13). This decline in pediatric anti-reflux volume parallels a near 2-fold (43% reduction) in the total cases reported by the graduating pediatric surgery fellows over the same time frame. Pediatric fundoplication being an advanced minimally invasive procedure has been a workhorse procedure allowing pediatric surgical trainees to gain the skills translatable to other minimally invasive procedures. This considerable decline in fundoplication experience is a difficult experience to replace, however identification of the best strategy for optimal patient outcomes obviously remains paramount.

There are some key limitations of this study that bear discussion. There are inherent biases and limitations from the retrospective nature of the study itself. As is frequently reported by other centers and detailed above at our institution there is no predefined investigative pathway for evaluation of GER pre-operatively. While most patients considered high risk for GER pre-operatively (on the basis of our preoperative evaluation) were initially managed in the GT with ARP approach, in the absence of a discrete qualifier suggestive of the absence of pathological GER at the index operation some patients may have had a degree of GER in the GT alone cohort. Despite these limitations we believe that this comparison group is a fairly accurate representation of the real-world decision making when evaluating a patient in need of long-term enteral feeding access. Carefully designed prospective randomized controlled design studies among pediatric patients are needed to validate the utility of the number of modalities previously discussed to develop an accurate method of predicting which patients really need an ARP at the time of index operation for long-term enteral feeding access. All surgeons in this report performed ARP in a similar manner, however, the choice of technique can have implications on outcomes and standardization of technique would be important if a prospective study is undertaken. While awaiting that information it appears that most patients can be fairly safely managed with a GT alone realizing that little is lost with this approach.

## Conclusion

When evaluating patients in need of long-term enteral feeding access, accurately identifying patients likely to benefit from a concomitant anti-reflux procedure remains challenging. A considerable proportion (20%) of GT patients needed a subsequent procedure despite most high-risk patients having already undergone an anti-reflux procedure at the outset. Additional prospective studies are needed and diagnostic approaches delineated that will help identify the patients most likely to benefit from an anti-reflux procedure at the same time feeding access is obtained. While such data is awaited since the overall rate of complications was similar, an initial GT alone approach can be considered reasonable. These data may also be helpful when discussing with families and the referring teams.

## Data Availability Statement

The raw data supporting the conclusions of this article will be made available by the authors, without undue reservation.

## Ethics Statement

The studies involving human participants were reviewed and approved by University of Florida. Written informed consent from the participants' legal guardian/next of kin was not required to participate in this study in accordance with the national legislation and the institutional requirements.

## Author Contributions

FK, KN, and SI: study conception and design. KN and SI: critical revision. FK and AH: drafting of the manuscript. FK, KN, AH, and SI: analysis and data interpretation. KN: data acquisition. All authors contributed to the article and approved the submitted version.

## Conflict of Interest

The authors declare that the research was conducted in the absence of any commercial or financial relationships that could be construed as a potential conflict of interest.

## Publisher's Note

All claims expressed in this article are solely those of the authors and do not necessarily represent those of their affiliated organizations, or those of the publisher, the editors and the reviewers. Any product that may be evaluated in this article, or claim that may be made by its manufacturer, is not guaranteed or endorsed by the publisher.
